# Porcine Acellular Dermal Matrix: An Alternative to Connective Tissue Graft—A Narrative Review

**DOI:** 10.1155/2021/1652032

**Published:** 2021-09-06

**Authors:** Shashi Dadlani

**Affiliations:** Section of Periodontology, Faculty of Health Sciences, University Clinic of Dentistry, Oviedo university, Spain

## Abstract

Porcine acellular dermal matrix has recently been introduced in dentistry as an alternative to the gold standard connective tissue graft especially for the use in gingival recession treatments and soft tissue augmentation in implant surgery. Connective tissue grafts are inconvenient and require a second surgical site leading to greater morbidity, longer surgical procedures, and a more painful postoperative phase for the patient. Other options such as allografts have ethical concerns and are less available in Europe. Thus, dental professionals have sought other techniques and materials. Porcine acellular dermal matrix results in periodontal recession treatment with a gain in recession coverage as well as increased keratinized tissue and soft tissue augmentation. This leads to more keratinized mucosa and greater tissue thickness. Many studies have been published using collagen matrices, but a few strictly use porcine acellular dermal matrix, which have been studied in prospective randomized clinical trials with a large number of patients and longer follow-up periods (more than 5 years). Nevertheless, more data are needed to confirm that the porcine acellular dermal matrix is a suitable alternative although its favourable results to date suggest a positive future.

## 1. Introduction

The porcine acellular dermal matrix (PADM) is a collagen matrix known commercially as mucoderm (Botiss Dental, Berlin, Germany). It has recently been introduced in dentistry as a substitute for the gold standard connective tissue graft (CTG) in periodontal and implant plastic surgery [1–4]. CTGs have limited availability especially when treating multiple gingival recessions. The need for a second surgical site can lead to postoperative pain and increased patient discomfort. There is also a greater risk of bleeding as well as a greater risk of damage to the branches of the palatine artery. Other disadvantages include a longer surgical procedure, greater risk of mucosal necrosis, and the need for hypnosis or anaesthesia especially when extracted from the palate. This has prompted periodontists to employ alternative materials [5–9].

Alternatives include allografts (AlloDerm) which is an acellular dermal matrix originally introduced to treat burn patients in the year 1992 and has since been used in medical and dental reconstructive surgery as a substitute for CTG with no risk of rejection or disease transmission [10, 11]. It consists of an allogenic freeze-dried connective tissue matrix, which has its epidermal layer and cellular components removed keeping its native three-dimensional structure composed of collagen and key extracellular matrix components including fibronectin, proteoglycans, and vascular channels which support cell migration and capillary proliferation. Such materials have raised ethical concerns due to their human origin, and most European countries including Spain have imposed heavy restrictions increasing the popularity of alternatives such as PADM [12–14].

PADM has greater availability than ADM; it can be harvested in bigger quantities. PADM consists of three-dimensional sterilized pure collagen types I and III and elastin, which is a stable tissue matrix derived from porcine dermis without being posteriorly cross-linked artificially or put under any other chemical treatment [15]. PADM also passes through a disinfecting process to eliminate all antigenic and tissue rejection constituents such as noncollagenous proteins and cells as well as bacteria and viruses from the dermis [16].

In vitro and in vivo investigations proved that PADM can increase growth and proliferation of human gingival fibroblasts, osteoblasts, and endothelial cells revealing a capacity for significant revascularization of the collagen structure during early healing [16–18]. Angiogenesis and the development of blood vessels are fundamental for nutrition, oxygen supply, immune cells, mesenchymal stem cells, and growth factors early in the healing period [19]. Lin et al. showed that PADM facilitates migration, adhesion, and proliferation of periodontal ligament cells and human oral fibroblasts [20].

## 2. Comparison with Connective Tissue Graft in Recession Treatment

One of the main reasons that collagen matrices have been introduced in dentistry is to avoid using connective tissue grafts for the motives mentioned earlier in this article. Many articles have been published comparing the gold standard with collagen matrices such as PADM for gingival recession treatment (see Table 1). This can increase the width of the attached gingiva on teeth and soft tissue augmentation during implant therapy.

Schmitt et al. published a 10-month preclinical study comparing CTG and PADM in beagle dogs and concluded that PADM was not statistically inferior for tooth volume augmentation of keratinized gingiva than CTG [3].

Cieślik-Wegemund et al. published a six-month randomized clinical trial comparing PADM with CTG using the tunnel technique to treat Miller Class I and II periodontal recessions stating an adequate recession coverage in patients treated with collagen matrix. There was a gain in keratinized tissue similar to CTG although there were fewer complete recession coverages [2]. Favourable results were also observed in a 12-month follow-up by Cosgarea et al. Evaluating PADM in multiple gingival recession treatments using a modified coronally advanced technique also included Miller Class III recessions [21].

In 2019, Gurlek et al. compared PADM and CTG and combined these materials with a modified coronal advanced flap in the treatment of multiple recessions of Miller class I and II [1, 22]. Both procedures were very effective during the 18-month follow-up, although CTG resulted with a higher gain in keratinized tissue. PADM suffered greater tissue shrinkage and a higher probing depth compared to CTG [23].

In contrast, Pietruska et al. compared the results of both procedures in the treatment of multiple gingival recessions of Miller Class I and II in the mandible via a modified coronal advanced tunnel technique (MCAT) [24, 25]. The mean root coverage data on PADM was 53.20% and 83.10% with the CTG after one year. Complete recession coverage was 20% for PADM and 67% in CTG. The results favour CTG, but these statistical results do not fundamentally influence clinics.

Rakasevic et al. studied 20 patients with multiple adjacent gingival recessions comparing CTG and PADM. They found no significant differences in clinical and aesthetic outcomes using MCAT recorded for 6 and 12 months; however, the mean root coverage was statistically higher in patients treated with CTG. Twice as many patients presented complete root coverage when treated with CTG after 12 months [26].

Similar results between outcomes of multiple gingival recession using CTG and PADM were found in a 20-patient study using a coronal advanced flap (CAF) technique. Increased keratinized tissue, reduction in recession, and complete recession coverage was achieved in both cases. However, as seen in previous studies, more complete recession coverage was obtained in treatments with CTG than PADM especially when keratinized tissue is rare [4, 27].

There are few long-term studies using PADM as a collagen matrix in gingival recession treatment. Cosgarea et al. continued a study initiated in 2016 on 12 patients with a 12-month follow-up mentioned earlier in this article, but extended the follow-up period for four years with a smaller sample of 9 patients. The results demonstrated that the mean recession coverage after 4 years was significantly lower than after 12 months but higher than baseline. Other measured parameters improved after surgery such as recession depth, recession width, width of keratinized tissue and attached gingiva, probing depth, and clinical attachment level [28].

A recent publication by Vincent Bugnas et al. comparing CTG with PADM also using the MCAT technique in 12 patients in a 12 month follow-up proved in this case that CTG obtained better results for mean recession coverage 80.6% ± 23.7% compared to 68.8% ± 23.4% in PADM and also in complete recession coverage 48.7% ± 6.8% with CTG and 24.3% ± 8.2% using PADM. In this study, all parameters that were recorded were in favour of the CTG procedures. However, as we mentioned earlier, they also demonstrated a reduced morbidity and less postoperative pain [29].

The latest publication comparing CTG and PADM this time using the CAF procedure in a study carried out in Brazil on 42 patients divided equally into the control (CTG) group and the test group (PADM), resulting in 18 patients in each group and on a 12 month follow-up, also resulted in better results for CTG specially in MRC in which CTG obtained 91.79 ± 10.1 in comparison with the test group result which was much lower 80.19 ± 16.3. Also, keratinized tissue gain was higher in the control group 0.99 ± 1.23 compared with 0.63 ± 0.83 in the test group [30].

According to recent literature, CTG provides greater gingival margin stability than PADM over time. In a review of 2554 gingival recessions on 1864 patients carried out in 2019, CTG provided a higher recession coverage than PADM and a less tendency for gingival recession recurrence [31].

In summary, gingival recessions have been treated with PADM and showed generally favourable results. However, it is still early to use PADM as a substitute for autogenous connective tissue grafts in mucogingival surgery; more studies are needed to defend this position with longer-term evaluations.

## 3. Comparison with Connective Tissue Graft in Implant Surgery for Soft Tissue Augmentation

Beyond gingival recession, PADM is also used in implant surgery for soft tissue augmentation [32]. It can increase keratinized mucosa (KM) width and thickness. Although there is no official consensus with specific guidelines for the requirement of KM or a minimum volume of peri-implant mucosa required to prevent peri-implant disease, there are many publications clinically proving that the lack of KM will increase the levels of plaque deposits around implants [33, 34].

Recent studies have also demonstrated the importance of having more than 2 mm of KM around implants to reduce the risk of peri-implant disease [35]. Publications by Linkevicuis et al. in 2009 showed lower marginal bone loss in implants that had more than 2 mm of KM around them [36]. Other more recent studies proved the need for a minimum of 2 mm of KM to minimize the risk of peri-implantitis [37, 38].

PADM is also being used as an alternative for soft augmentation procedures in implant dentistry substituting the gold standard (CTG). A clinical study published in 2014 by Nocini et al. tested PADM in an extensive keratinized tissue augmentation with deepening of the vestibule. They collected data over different periods: 9 days, 14 days, as well as 1 and 2 months. There was noticeable augmentation of KM around implants and deepening of the vestibule; some buccal KM contraction was observed [39].

Considerable KM gain in width was also observed. One prospective pilot cohort study by Papi et al. used PADM in peri-implant soft tissue augmentation on a second-stage surgery with a small sample of 12 patients. This study did not include a control group using CTG. The KM width increased from 1.35 ± 0.32 mm to 7.86 ± 3.22 mm after a month and 5.67 ± 2.12 mm after a year with an increase of 72.13%. Shrinkage was also observed especially from the first month to the 12th month [40].

Later, the same authors published an article in which a prospective cohort study of a two-year follow-up period in an early implant placement surgery and a concomitant peri-implant augmentation was performed. PADM was combined with synthetic bone in the aesthetic zone. Keratinized mucosa width and gingival thickness were assessed in different periods of 1, 3, 6, 12, and 24 months. Both KM width and gingival thickness increased from baseline to the first month and then decreased from the 1st to the 12th month and remained stable from 12 to 24 months. After 24 months, a 1.94 ± 0.05 mm gain was observed in gingival thickness and a 1.60 ± 0.11 mm gain in KM width compared to baseline [32].

Another important value measured after soft augmentation procedures with PADM as mentioned earlier is soft tissue thickness. As early as 2015, a 6-month follow-up and a sample of 27 patients by Zafiropoulous et al. concluded that PADM leads to a significant increase in soft tissue thickness of 1.06 mm. Although this study had a control group without using any type of graft, previous publications state that the use of CTG increases tissue thickness to an average of 1.2–1.75 mm, which is higher than PADM [41, 42]. Excellent histological integration and substitution of soft tissue was observed; hence, PADM can be an alternative to CTG [43].

Similar results were observed in soft tissue thickness gain in a longer follow-up of 12 months by Stefanini et al. This study followed 10 patients utilizing the coronal advanced flap surgical technique put together with PADM. There was a 1.2 ± 0.18 mm gain of tissue thickness in the aesthetic zone one year after the final restoration. The authors also found compliance with aesthetic-functional requirements in implant sites. However, this study had no control group [44].

Papi et al. also reported on 12 patients a year later in 2020, where PADM was used for soft tissue augmentation. A second-stage surgery with a 12-month follow-up also demonstrated a gain in tissue thickness this time with a mean gain of 1.25 mm. This is a slightly higher figure (mucosal thickness) than in Stefanini et al. This recent study contrasts with previous ones and also included a three-dimensional volumetric measurement analysis. The first one was published on the buccal contour. The area measured for volume analysis showed a 51.501 mm^3^ mean gain and 23.31% shrinkage from the first month after PADM was placed [45].

Schmitt et al. also compared CTG and PADM in this case with a 3D follow-up and including a test and control group with increasing CTG-measured tissue thickness and volume. The volume increase after the six-month period was 19.56 ± 8.95 mm^3^ for PADM and 61.75 ± 52.69 mm^3^ for CTG. In the case of tissue thickness in the area of the buccal contour, PADM increased thickness by 0.30 ± 0.16 mm versus CTG 0.80 ± 0.61 mm both after 6 months. The authors concluded that CTG had better results than PADM for both values (soft tissue thickness and volume increase) [46]. A similar study published in 2020 on 15 patients during a three-year follow-up also measured the soft tissue volume at the buccal aspect. This used implant molar sites with a digital volumetric analysis and strictly used PADM as a collagen matrix. The mean increase in “buccal soft tissue profile” or volume increase was 1.17 mm (76.5%) after three years. Volume was measured in a specific area in the buccal site at different periods of time: before surgery, immediately after surgery, and after 3 months, 12 months, and 36 months. After surgery, the mean volume increase was 1.53 mm, and after 3 months, it decreased to 1.02 mm due to shrinkage and PADM resorption [40]. After this, a 0.66 mm gain was observed that the authors attribute to the permanent restoration placement pushing the buccal soft tissue [47].

The difference with previous studies such as Stefanini and Papi is that the PADM was placed the same day during the implant surgery. Moreover, in contrast to previous studies like Zafiropoulos and Stefanini which had shorter follow-up periods, these studies carried out measurements with less accurate methods such as transmucosal probing with instruments used in endodontics or anesthetic needles. There were no intermediate measurements after surgery.

A final study, recently published, compared CTG and PADM regeneration techniques to gain keratinized tissue thickness carried out in pigs clinically and histologically examined in three different spans of time: 15 days, 45 days, and 90 days. This showed that the group with PADM had average keratin layer thickness values on top of the CTG group [48]. From 15 to 45 days, there was notable resorption as in previous studies such as Eeckhout et al. and Stefanini et al.

Another important consideration is that CTG when extracted can be of different thicknesses in contrast to PADM which has the same thickness. In further studies, it may be necessary to standardize completely and introduce the same thickness of CTGs for examination.

A summary of significant research studies comparing CTG and PADM in soft tissue augmentation procedures measuring keratinized mucosa and soft tissue thickness is presented in Table 2.

## 4. Conclusion

Randomized clinical trials have been published using strictly porcine acellular dermal matrix as a collagen matrix in dental recession treatment or for soft tissue augmentation in implant surgery. Fewer prospective long-term (>5 years) longitudinal studies exist. The porcine acellular dermal matrix is used in periodontal plastic (gingival recession treatment) and implant surgery and has favourable results as shown in several publications. According to the consensus of the publications reviewed, PADM while providing a benefit in root coverage procedures, it fell short of the outcomes achieved with CTG. At implant sites, soft tissue augmentation procedures offered a favourable outcome similar to CTG for increasing soft tissue thickness.

More studies are needed to compare PADM and CTG using three-dimensional techniques via STL and CBCT superimposition. It is still too early to decide on PADM versus the gold standard (CTG). More evidence is needed including longer trials and larger patient cohorts. PADM has many advantages especially in terms of reduced morbidity and reduced surgical times.

## Figures and Tables

**Table 1:** Porcine acellular dermal matrix in recession treatment: comparing mean recession coverage and keratinized tissue width between procedures carried out with CTG or PADM.

Authors	Sample	Type of study	Follow-up time	Surgical procedure	Mean recession coverage (%)	Keratinized tissue width gain (mm)
Schmitt et al. [3]	8 dogs	Preclinical study	10 months	Tunnel technique	MRC not registered	KTW not registered
Cieślik-Wegemund et al. [2]	28 patients	Randomized clinical trial	6 months	Tunnel technique	CTG: 95% PADM: 91%	CTG: 1.0 PADM: 0,8
Scuelan et al. [21]	12 patients	Case series	12 months	MCAT	PADM: 73.20 ± 27.71% No CTG control	PADM: 0.69 ± 0.51 No CTG control
Gürlek et al. [1]	12 patients	Split-mouth randomized clinical trial	18 months	CAF	MRC not registered	CTG: 0.51 ± 0.60 PADM: 0.32 ± 0.52
Pietruska et al. [24]	29 patients	Randomized clinical trial	12 months	MCAT	CTG: 83.10% PADM: 53.20%	CTG: 2.78 ± 1.53 PADM: 0.52 ± 0.65
Rakasevic et al. [26]	20 patients	Randomized clinical trial	12 months	MCAT	CTG: 2.96 ± 11.8% PADM: 1.71 ± 13.7%	CTG: 0.84±1 PADM: 0.85 ± 1.2
Maluta et al. [4]	15 patients	Split-mouth randomized clinical trial	6 months	CAF	CTG: 95.28 ± 6.89% PADM: 92.68 ± 7.35%	CTG: 0.91 ± 0.46 PADM: 0.74 ± 0.39
Cosgarea et al. [28]	9 patients	Case series	48 months	MCAT	PADM: 56.79 ± 27.53% No CTG control	PADM: 0.26 ± 0.72 No CTG control
Vincent Bugnas et al. [29]	12 patients	Random split mouth clinical trial	12 months	MCAT	CTG: 80.6 ± 23.7%, PADM: 68.8 ± 23.4%	CTG: 0.91 ± 0.46l PADM: 0.74 ± 0.39
Meza- Mauricio et al. [30]	42 patients	Randomized controlled clinical trial	12 months	CAF	CTG: 91.79 ± 10.1% PADM: 80.19 ± 6.3%	CTG: 0.99 + 1.23 PADM: 0.63 ± 0.83

**Table 2:** Soft tissue augmentation in implant sites: comparing keratinized mucosa and soft tissue thickness in procedures using PADM or CTG.

Authors	Sample	Study design	Follow-up time	Keratinized mucosa width gain (mm)	Soft tissue thickness gain (mm)
Zafiropoulos et al. [43]	27 patients	Prospective, randomized examiner- blinded controlled clinical study	6 months	Not registered	PADM: 1.06 CTG: no CTG control
Papi and Pompa [17]	12 patients	Prospective pilot cohort study	12 months	PADM: 4.32	Not registered
Stefanini et al. [44]	10 patients	Case series	12 months	0.65 ± 0.41 No CTG control	PADM: 1.2 ± 0.18 No CTG control
Papi et al. [32]	20 patients	Prospective cohort study	24 months	PADM:1.60 ± 0.11 No CTG control	PADM: 1.94 ± 0.05 No CTG control
Eeckhout et al. [47]	15 patients	Prospective case series	36 months	Not registered	PADM: 0.66 No CTG control
Schmitt et al. [46]	14 patients	Controlled clinical trial	6 months	Not registered	CTG: 0.80 ± 0.61 PADM: 0.30 ± 0.16
Papi et al. [45]	12 patients	Prospective cohort study	12 months	Not registered	PADM: 1.25 No CTG control

## Data Availability

All data in the article are cited in references.
